# Genome-Wide Survey and Analysis of Microsatellite Sequences in Bovid Species

**DOI:** 10.1371/journal.pone.0133667

**Published:** 2015-07-21

**Authors:** Wen-Hua Qi, Xue-Mei Jiang, Lian-Ming Du, Guo-Sheng Xiao, Ting-Zhang Hu, Bi-Song Yue, Qiu-Mei Quan

**Affiliations:** 1 College of Life Science and Engineering, Chongqing Three Gorges University, Chongqing, 404100, China; 2 College of Environmental and Chemistry Engineering, Chongqing Three Gorges University, Chongqing, 404100, China; 3 Key Laboratory of Bio-resources and Eco-environment (Ministry of Education), College of Life Sciences, Sichuan University, Chengdu, 610064, China; 4 School of Life Sciences, China West Normal University, Nanchong, 637009, China; University of Minnesota, UNITED STATES

## Abstract

Microsatellites or simple sequence repeats (SSRs) have become the most popular source of genetic markers, which are ubiquitously distributed in many eukaryotic and prokaryotic genomes. This is the first study examining and comparing SSRs in completely sequenced genomes of the Bovidae. We analyzed and compared the number of SSRs, relative abundance, relative density, guanine-cytosine (GC) content and proportion of SSRs in six taxonomically different bovid species: *Bos taurus*, *Bubalus bubalis*, *Bos mutus*, *Ovis aries*, *Capra hircus*, and *Pantholops hodgsonii*. Our analysis revealed that, based on our search criteria, the total number of perfect SSRs found ranged from 663,079 to 806,907 and covered from 0.44% to 0.48% of the bovid genomes. Relative abundance and density of SSRs in these Bovinae genomes were non-significantly correlated with genome size (Pearson, *r* < 0.420, *p* > 0.05). Perfect mononucleotide SSRs were the most abundant, followed by the pattern: perfect di- > tri- > penta- > tetra- > hexanucleotide SSRs. Generally, the number of SSRs, relative abundance, and relative density of SSRs decreased as the motif repeat length increased in each species of Bovidae. The most GC-content was in trinucleotide SSRs and the least was in the mononucleotide SSRs in the six bovid genomes. The GC-contents of tri- and pentanucleotide SSRs showed a great deal of similarity among different chromosomes of *B*. *taurus*, *O*. *aries*, and *C*. *hircus*. SSR number of all chromosomes in the *B*. *taurus*, *O*.*aries*, and *C*. *hircus* is closely positively correlated with chromosome sequence size (Pearson, *r* > 0.980, *p* < 0.01) and significantly negatively correlated with GC-content (Pearson, *r* < -0.638, *p* < 0.01). Relative abundance and density of SSRs in all chromosomes of the three species were significantly negatively correlated with GC-content (Pearson, *r < *-0.333, *P *< 0.05) but not significantly correlated with chromosome sequence size (Pearson, *r *< -0.185, *P* > 0.05). Relative abundances of the same nucleotide SSR type showed great similarity among different chromosomes of *B*. *taurus*, *O*. *aries*, and *C*. *hircus*.

## Introduction

Microsatellites, also known as simple sequence repeats (SSRs), are tandem repetitions of 1–6 base pair (bp) nucleotide motifs of DNA sequences [1]. SSRs have been developed into one of the most popular sources of genetic markers owing to their high reproducibility, multi-allelic nature, co-dominant mode of inheritance, abundance, and wide genome coverage [2], which have been widely employed in population genetics, phylogenetics, genetic mapping, linkage, and kinship relationships [3]. Although SSRs are ubiquitously distributed throughout eukaryotic and prokaryotic genomes [4, 5], and are even in the small virus genomes [6], the density and distribution of SSRs vary markedly across whole genomes [7]. SSR loci have a high mutation rate (10^−4^ to 10^−3^) [8] which resulted in high heterozygosity and the presence of multiple alleles [9]. SSRs have been found in both coding and non-coding regions [10], which are supposed to serve a functional role affecting gene regulation, transcription, protein function, and genome organization [11–13].

However, the conventional methods of generating SSR markers from genomic libraries are challenging, costly, labor consuming and time consuming [14], which are being replaced rapidly by *in silico* mining of SSR sequences from DNA-sequence databases [15–16]. More recently, the availability of enormous genome sequences for a wide range of organisms, together with new methodological developments of *in silico* mining of SSRs, has accelerated research aimed at understanding the origin and functions of SSRs and at searching for new applications, and will certainly promote the study of genomic distribution of SSRs in the eukaryotic and prokaryotic genomes. The possibility of cross-amplification of SSR markers in closely related species has increased their usefulness extremely. Therefore, scientific and reasonable microsatellite mining not only helps in addressing biological questions but also facilitates better exploitation of microsatellites for various applications.

The recent completion of genome sequencing projects has provided new opportunities to evaluate and compare the distribution of SSRs at the genomic level. There are now six bovid species with complete sequencing: *Bos taurus*, *Bos mutus*, *Bubalus bubalis*, *Ovis aries*, *Capra hircus*, and *Pantholops hodgsonii*. The complete genomes of these six species will facilitate the study of the mechanism of their secondary metabolism and provide an opportunity to scan the entire genome for SSR discovery. No genome-wide survey of SSRs is available for the Bovidae, hence we report here the first survey and comparative analysis of SSRs, and reveal consistent patterns of the distribution, abundance, density, and diversity of different SSRs in the genomes of six species of the Bovidae. We compared the relative abundance and density of mono- to hexanucleotide SSRs among the six bovid genomes. The distributions of perfect mono- to hexanucleotide SSRs on all chromosomes were also compared in three of the species: *B*. *taurus*, *O*. *aries*, and *C*. *hircus*. Though guanine-cytosine (GC) content has been reported to have a certain influence on the occurrence and polymorphic nature of SSRs [7,17], which is seldom systematically studied. So the GC-content of SSRs was systematically analyzed in these bovid genomes. Lastly, primers were designed for the identified SSR loci in order to provide the material basis for the future development of a wide range of SSR markers in the bovidaes. Our study will serve to establish the SSR distribution patterns among closely/less closely related species and contribute to their future use as molecular markers.

## Materials and Methods

### 2.1 Genome sequences

At the time of this study, only six species of the Bovidae were known to have complete genome sequences according to the genomic resources of the NCBI (National Center of Biotechnology Information). So we selected these six genome sequences as samples to analyze the SSR distributions in the genomic level. All the genome sequences were downloaded in FASTA format from the GenBank (http://www.ncbi.nlm.nih.gov). The species, genome size, the GC-content, etc., have been summarized in Table 1. The genome size ranged from ~2587.51 Mb (*O*. *aries*) to 2983.31 Mb (*B*. *taurus*).

**Table 1:** Overview of the six bovid genomes.

Parameters	*B*. *taurus*	*B*. *mutus*	*Bu*. *bubalis*	*O*. *aries*	*C*. *hircus*	*P*. *hodgsonii*
Genome size (Mb)	2983.31	2645.15	2836.15	2587.51	2635.87	2696.89
GC-content (in %)	41.81	41.71	42.11	41.79	41.75	42.01
# of SSRs	806,907	716,360	774,309	682,891	677,017	663,079
Relative abundance (#/Mb)	270.48	270.83	273.01	263.92	256.85	245.87
Total length of SSRs (bp)	14,270,305	12,423,719	13,556,032	12,412,553	12,134,448	11,937,706
Relative density (bp/Mb)	4783.37	4696.80	4779.73	4797.11	4603.62	4426.48
Genome SSRs content (%)	0.48	0.46	0.48	0.48	0.46	0.44

### 2.2 SSRs identification and investigation

SSRs were identified and localized using the software MSDB (Microsatellite Search and Building Database) downloaded at https://code.google.com/p/msdb/ [18], which is a Perl program providing a user-friendly interface for identification and building databases of SSRs from complete genome sequences. SSRs can be grouped into six categories: (1) pure or perfect (P) SSRs, (2) interrupted perfect (IP) SSRs, (3) compound (CD) SSRs, (4) interrupted compound (ICD) SSRs, (5) complex (CX) SSRs, and (6) interrupted complex (ICX) SSRs [19–20]. MSDB has two search modes: A ‘perfect search mode’ is used to search perfect SSRs or pure SSRs and an ‘imperfect search mode’ is used to search the six categories of SSRs mentioned above [18]. In order to search a sequence for perfect SSRs, the definition of the minimum repeat number is an important criterion. Since bovid species have very large genomes, relatively systemic search criteria were adopted in this study: The parameters for minimum repeat numbers were set as 12, 7, 5, 4, 4, 4 for mono-, di-, tri-, tetra-, penta-, and hexanucleotide SSRs, respectively [18]. The maximum distance allowed between any two SSRs (dMAX) was 10 bp; other parameters were set as default. In this study, repeats with unit patterns being circular permutations and/or reverse complements of each other were grouped together as one type for statistical analysis [21–22]. For example, ACT denotes ACT, CTA, TAC, TGA, GAT and ATG in different reading frames or on the complementary strand. For tetranucleotide and hexanucleotide repeats, combinations representing perfect di- and trinucleotide repeats were filtered from the final counts, for example, a (ACAC)_9_ was considered as a (AC)_18_ dinucleotide and not as a tetranucleotide repeat. The combinations of SSRs for this study will help to give a better understanding of the total occurrence of SSRs, and their genomic locations will be very useful in selecting SSRs representative of similar repeat classes from different genomic locations as potential markers.

To facilitate the comparison among different repeat categories or motifs, we used relative abundance, which means the number of SSRs per Mb of the sequence analyzed, and relative density, which means the length (in bp) of SSRs per Mb of the sequence analyzed [18, 23]. These total numbers have been normalized either as relative abundance or relative density to allow comparison among genome sequences of different sizes. The relative abundance and density on each chromosome was calculated by dividing the total chromosome length by each nucleotide SSR. Primer pairs for the identified SSR loci were designed using the Primer 3 software implemented in the MSDB using default parameters.

### 2.3 Statistical analysis

All data analyses were performed using SPSS version 18.0 and followed standard procedures. The Pearson test was used to reveal the correlation between two variables, including relative abundance, relative density, genome size, GC-content, and chromosome sequence size. Student’s t-test was used to compare means of two groups.

## Results

### 3.1 The number, relative abundance and density of SSRs in bovid genomes

**Fig 1:**
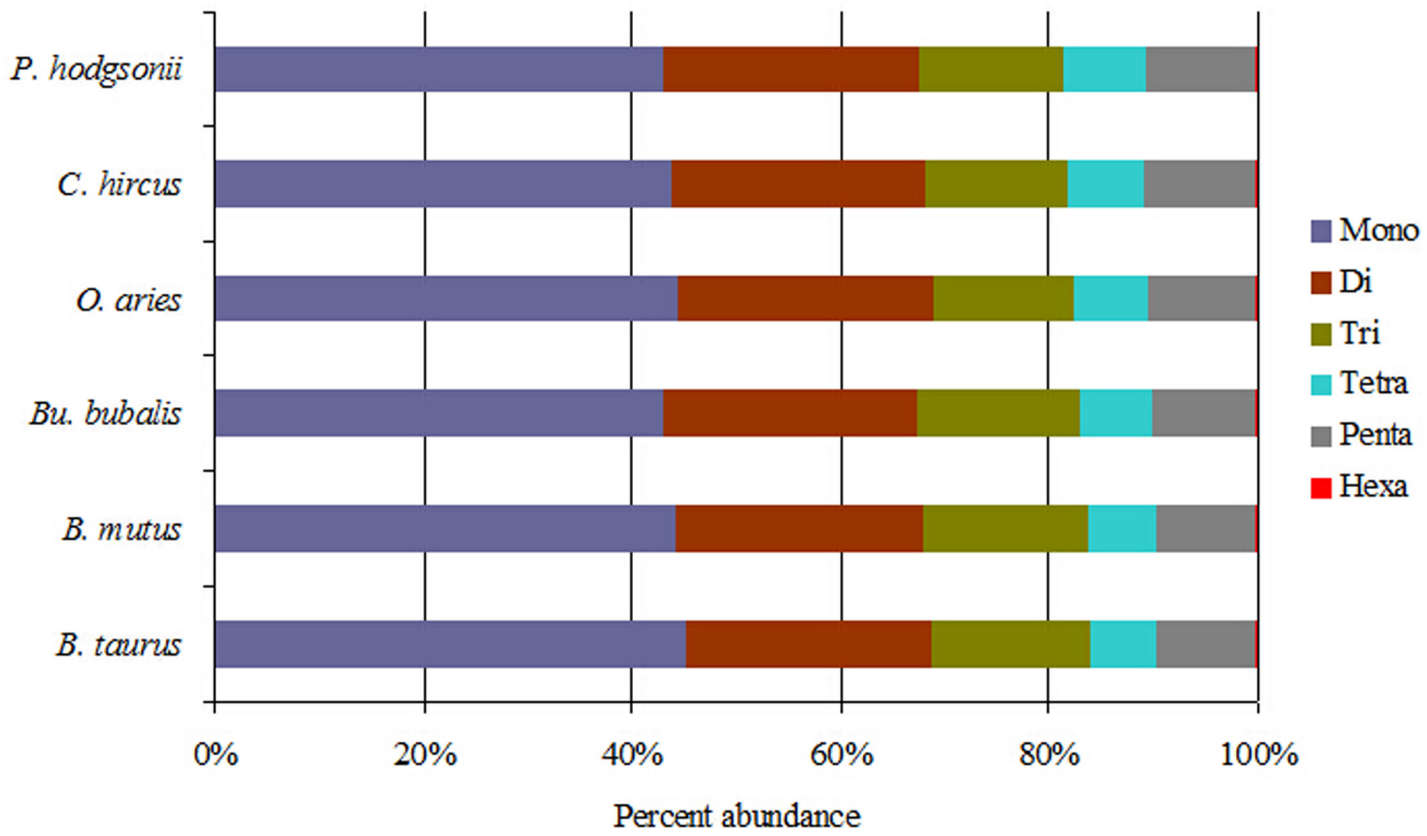
The distribution of perfect SSRs in the six bovid genomes. Percentages were calculated according to the total number of each perfect SSR category divided by the total number of SSRs for that organism.

The six categories of SSRs were found in each of these bovid genomic sequences by using computer software MSDB for a genome-wide scan (Table 2). P-SSRs was the most abundant type in these bovid species, followed by the pattern: CD-SSRs > ICD-SSRs > IP-SSRs > ICX-SSRs > CX-SSRs (Table 2). The relative abundances of the same SSR types showed great similarity in the Bovinae species and also in the Caprinae species. The number, relative abundance and density of perfect mono- to hexanucleotide repeat types across these species genomes are presented in Table 3. Results here indicated that the number, relative abundance, and density of the same repeat type of perfect SSRs (mono- to hexanucleotides) showed great similarity in the six bovid species. Perfect mononucleotide SSRs were the most abundant category, followed by the pattern: perfect di- > tri- > penta- > tetra- > hexanucleotide SSRs (Table 3). The proportion of mono- to hexanucleotide SSRs was very similar in the six bovid genomes (Fig 1). Mononucleotide SSRs were the maximum ratio, accounting for 43.02% ~ 45.33% of all of the SSRs, followed by the dinucleotide SSRs, whereas trinucleotide SSRs were the third most frequent. The proportion of pentanucleotide SSRs was more than that of tetranucleotide SSRs and hexanucleotide SSRs was the minimum percentage. There were non-significant differences in these parameters between Bovinae and Caprinae genomes (*t*-test, *p* > 0.05).

**Table 2:** Relative abundance of the six categories of SSRs in the bovid genomes.

Type	*B*. *taurus*	*B*. *mutus*	*Bu*. *bubalis*	*O*. *aries*	*C*. *hircus*	*P*. *hodgsonii*
CD-SSRs	4.03	3.42	3.92	3.68	3.39	3.44
CX-SSRs	0.15	0.13	0.14	0.15	0.11	0.15
ICD-SSRs	2.17	2.04	2.21	2.54	2.32	2.17
ICX-SSRs	0.40	0.34	0.37	0.52	0.41	0.37
IP-SSRs	1.37	1.47	1.39	2.4	1.86	1.55
P-SSRs	253.52	254.96	256.28	243.66	239.9	229.59

Note: Compound, CD; interrupted compound, ICD; complex, CX; interrupted complex, ICX; Perfect, P; interrupted perfect, IP.

**Table 3:** Number, abundance and density of mono- to hexanucleotide repeat type in the bovid genomes.

Repeat type		*B*. *taurus*	*B*. *mutus*	*Bu*. *bubalis*	*O*. *aries*	*C*. *hircus*	*P*. *hodgsonii*
Mono-	# SSRs	365,798	316,378	333,806	303,318	297,036	285,233
	Abundance (#/Mb)	122.61	119.61	117.7	117.22	112.69	105.76
	Density (bp/Mb)	1829.58	1759.18	1767.92	1750.45	1677.22	1537.07
Di-	# SSRs	187,846	171,526	187,499	167,117	164,986	163,312
	Abundance (#/Mb)	62.97	64.85	66.11	64.59	62.59	60.56
	Density (bp/Mb)	1380.85	1324.79	1364.80	1436.86	1350.32	1326.49
Tri-	# SSRs	123,674	112,936	121,982	91,709	92,073	90,696
	Abundance (#/Mb)	41.46	42.70	43.01	35.44	34.93	33.63
	Density (bp/Mb)	719.46	744.70	737.23	653.50	626.53	617.95
Tetra-	#SSRs	51,615	46,006	52,793	49,851	49,943	52,900
	Abundance (#/Mb)	17.30	17.39	18.61	19.27	18.95	19.62
	Density (bp/Mb)	298.90	301.74	325.73	353.53	341.88	361.72
Penta-	# SSRs	76,182	68,428	76,442	69,111	71,036	69,509
	Abundance (#/Mb)	25.54	25.87	26.95	26.71	26.95	25.77
	Density (bp/Mb)	537.95	554.38	566.31	582.83	587.20	568.36
Hexa-	# SSRs	1,792	1,086	1,787	1,785	1,943	1,429
	Abundance (#/Mb)	0.60	0.41	0.63	0.69	0.74	0.53
	Density (bp/Mb)	16.63	12.01	17.74	19.95	20.48	14.88

It is amazing to find that the number of SSRs is closely positively correlated with genome size (Pearson, *r* = 0.898, *p* < 0.05) and but not significantly correlated with GC-content (Pearson, *r* < 0.185, *p* > 0.05) in these bovid genomes. Neither relative abundance nor relative density of SSRs in these bovid genomes was significantly correlated with genome size (Pearson, *r* < 0.420, *p* > 0.05) and GC-content (Pearson, *r <* −0.121, *p* > 0.05). For example, *B*. *taurus* (v4.6.1) has the longest genome sequence length of 2,983.31 Mb among all surveyed species, while, it is not as we hoped that has the highest SSR abundance and density (270.48 /Mb and 4,783.37 bp/Mb, respectively). Similarly, *O*. *aries* (v3.1) has the shortest genome sequence length of 2,587.51Mb, while it has the highest SSR density. The number, relative abundance and density of pentanucleotide SSR is more than that of tetranucleotide repeat types in these genomes. *B*. *taurus* and *Bu*. *bubalis* showed the largest number of pentanucleotides with 52,793 and 52,900 loci, respectively. *Bu*. *bubalis* and *C*. *hircus* have the highest and same relative abundance of pentanucleotides with 29.65 /Mb (Table 3), even though *B*. *taurus* has the lowest relative abundance (25.54 /Mb).

### 3.2 Diversity of SSRs in the bovid genomes

The most frequent motifs for different length varied with the different bovid species at the whole genome level (Table 4) and the chromosome level (S1 Table). Among mononucleotide repeat type, the motif (A)_n_ were predominant (over 93.27%), while (C)_n_ repeats were rare (less than 6.73%) in these bovid species genomes, with no obvious relation to the AT-richness of the genomes (Pearson, *r* < 0.160, *p* > 0.05). (AC)_n_, (AT)_n_ and (AG)_n_ were the three most frequent dinucleotide SSRs motifs, the three of which accounted for over 99% of all motifs of dinucleotide SSRs in each genome and each chromosome. In contrast, the (AC)_n_ motif was particularly dominant, the (AT)_n_ and the (AG)_n_ motifs were less abundant, and (CG)_n_ was the least frequent motifs found in any of the six genomes and each chromosome of *B*. *taurus*, *O*. *aries*, and *C*. *hircus*. In the trinucleotide repeat type, (ACG)_n_ and (AGC)n were the most frequent motifs, followed by the (AAC)_n_, (AAT)_n_ and (ACC)_n_ motifs in these bovid genomes and each chromosome of *B*. *taurus*, *O*. *aries*, and *C*. *hircus* (except for Y chromosome). The (CCG)_n_ motif was the least frequent in *B*. *mutus*, *O*. *aries*, *C*. *hircus*, and *P*. *hodgsonii* genomes, while the (AGT)_n_ motif was the least frequent in *B*. *taurus* and *Bu*. *bubalis* genomes. The most frequent tetranucleotide SSRs motif was found to be the (AAAT)_n_ unit, followed by the (AAAC)_n_ and (AAAG)_n_ motifs, and the (CCGG)_n_ motifs was the least frequent in the six Bovidae genomes and each chromosome of *B*. *taurus*, *O*. *aries*, and *C*. *hircus*. The richness of tetranucleotide repeats is less than that of mono- to trinucleotide repeat motifs in these genomes except for the (AAAT)_n_, (AAAC)_n_ and (AAAG)_n_ motifs. The most frequent motifs of mono- to tetranucleotide was more invariable, with the list of most frequent motifs becoming identical for each bovid species, and the most frequent penta- and hexanucleotide motifs appeared to be more variable among these species, and each genome displayed its own characteristic. Penta- and hexanucleotide SSRs have a great many motifs in all six genomes. The (AACTG)_n_ and (AGTTC)_n_ motifs were the two most frequent tetranucleotide repeat units in these species and each chromosome of *B*. *taurus*, *O*. *aries*, and *C*. *hircus*, and none of these single hexanucleotide motifs appeared to be shared by the six bovid species. The (AAACAA)_n_ motif was most frequent in *B*. *mutus*, *Bu*. *bubalis*, *O*. *aries*, and *C*. *hircus* genomes, whereas the (AAAGTG)_n_ motif was most frequent in *B*. *taurus* and *P*. *hodgsonii* genomes. The telomeric-like hexanucleotide (AACCCT)_n_ motif was also observed in all six genomes. The most frequent tetra- to hexanucleotide motifs appeared to be more variable between Bovinae and Caprinae species.

**Table 4:** The number, abundance, and density of the most frequent SSR motifs in the bovid genomes.

Repeat motif type		*B*. *taurus*	*B*. *mutus*	*Bu*. *bubalis*	*O*. *aries*	*C*. *hircus*	*P*. *hodgsonii*
A	# SSRs	355,536	310,492	326,082	282,896	283,296	274,802
	Abundance (#/Mb)	119.18	117.38	114.98	109.34	107.48	101.89
	Density (bp/Mb)	1782.09	1724.56	1726.58	1611.5	1585.57	1475.36
C	#SSRs	10,262	5,886	7,724	20,422	13,740	10,431
	Abundance (#/Mb)	3.44	2.22	2.73	7.89	5.21	3.87
	Density (bp/Mb)	47.49	34.63	41.34	138.94	91.65	61.72
AC	# SSRs	111,720	105,664	114,982	101,967	101,862	102,687
	Abundance (#/Mb)	37.45	39.94	40.54	39.4	38.64	38.07
	Density (bp/Mb)	835.2	836.19	884.77	916.92	883.37	856.58
AG	# SSRs	17,991	16,290	18,244	16,850	16,719	16,337
	Abundance (#/Mb)	6.03	6.15	6.44	6.51	6.34	6.06
	Density (bp/Mb)	108.84	113.63	115.13	135.35	122.57	121.58
AT	# SSRs	57,682	49,266	53,736	47,947	46,035	43,764
	Abundance (#/Mb)	19.33	18.63	18.95	18.53	17.46	16.23
	Density (bp/Mb)	434.37	373.14	361.77	382.47	342.19	345.22
AAC	# SSRs	9,039	8,864	9,610	9,877	9,874	8,638
	Abundance (#/Mb)	3.03	3.36	3.38	3.82	3.74	3.21
	Density (bp/Mb)	49.74	55.46	56.15	66.06	64.31	53.78
AAT	# SSRs	7,148	6,484	7,143	6,565	6,685	6,911
	Abundance (#/Mb)	2.39	2.45	2.52	2.54	2.54	2.56
	Density (bp/Mb)	39.32	39.98	41.71	43.34	43.08	43.07
ACC	# SSRs	4,040	4,336	4,301	4,017	3,797	3,672
	Abundance (#/Mb)	1.35	1.64	1.52	1.56	1.44	1.37
	Density (bp/Mb)	22.64	28.04	25.42	26.37	24.06	23.07
ACG	# SSRs	46,957	43,571	45,987	32,073	32,672	32,527
	Abundance (#/Mb)	15.74	16.48	16.21	12.39	12.39	12.06
	Density (bp/Mb)	275.9	287.74	280.2	234.55	226.52	229.39
AGC	#SSRs	47,388	42,783	46,687	32,669	33,248	32,674
	Abundance (#/Mb)	15.89	16.18	16.46	12.62	12.61	12.11
	Density (bp/Mb)	278.99	286.15	284.4	238.19	229.7	227.51
AAAC	# SSRs	8,655	7,441	8,920	9,215	8,811	8,667
	Abundance (#/Mb)	2.9	2.81	3.15	3.56	3.34	3.22
	Density (bp/Mb)	50.83	49.1	55.1	64.41	60.12	57.32
AAAG	# SSRs	4,781	3,800	4,271	4,597	4,451	4,695
	Abundance (#/Mb)	1.6	1.44	1.51	1.77	1.69	1.74
	Density (bp/Mb)	27.86	25.12	25.98	34.81	30.77	32.9
AAAT	#SSRs	13,331	12,057	14,505	13,116	13,866	15,461
	Abundance (#/Mb)	4.47	4.56	5.12	5.07	5.26	5.74
	Density (bp/Mb)	75.66	77.56	87.69	91.18	93.65	105.52
AAGT	# SSRs	2,129	2,110	2,210	2,069	2,031	2,172
	Abundance (#/Mb)	0.72	0.8	0.78	0.8	0.77	0.81
	Density (bp/Mb)	12.09	13.51	13.14	13.71	13.17	13.86
AATG	# SSRs	2,285	2,153	2,288	2,052	2,060	2,091
	Abundance (#/Mb)	0.77	0.82	0.8	0.8	0.78	0.77
	Density (bp/Mb)	12.99	13.84	13.64	13.6	13.32	13.36
AACTG	# SSRs	27,949	24,909	27,273	30,067	31,569	30,331
	Abundance (#/Mb)	9.37	9.41	9.62	11.62	11.97	11.24
	Density (bp/Mb)	196.83	202.44	201.53	252.94	260.71	247.43
AGTTC	# SSRs	27,045	24,862	26,597	29,884	30,603	30,581
	Abundance (#/Mb)	9.07	9.4	9.38	11.55	11.61	11.34
	Density (bp/Mb)	190.36	199.38	196.52	252.33	252.88	249.02

### 3.3 The GC-content of all perfect SSRs in the bovid genomes

The adenine-thymine (AT) and GC-content were calculated in perfect SSRs of bovid genomes. The results were shown in Table 5. From the results, we can know that except for the trinucleotide SSRs, the AT-content of the remaining nucleotide repeat types are more than the GC-content. Mononucleotide SSRs had the most AT-content (over 92.06%), followed by the pattern: tetra- > di- > penta- > hexanucleotide SSRs, and the least was in the trinucleotide SSRs (ranging from 40.11% to 42.68%) in the six bovid genomes. On the other hand, we analyzed the GC-content of SSRs in the bovid genomes. The results showed that the most GC-content is in the trinucleotide, ranging from 57.32% (*C*. *hircus*) to 59.89% (*B*. *taurus*), and the least is in the mononucleotide, ranging from 1.97% to 7.94% in these genomes. In contrast, the GC-content in all mononucleotide SSRs was significantly lower than that in entire genome, and the GC-content in the di- and tetranucleotide SSRs were also less than that in entire genome in these analyzed genomes, and the GC-content in the remaining SSRs was more than that in entire genome. In the bovid entire genome, the total AT-contents range from 71.44% to 73.78%, were significantly higher than the GC-content. Therefore, the AT-content of SSRs is very high in the bovid species.

**Table 5:** The AT- and GC-content of perfect SSRs in the bovid genomes.

Repeat type		*B*. *taurus*		*B*. *mutus*		*Bu*. *bubalis*		*O*. *aries*		*C*. *hircus*		*P*. *hodgsonii*	
		Length (bp)	%	Length (bp)	%	Length (bp)	%	Length (bp)	%	Length (bp)	%	Length (bp)	%
Mono-	A + T	5,316,524^a^	97.40^b^	4,561,708	98.03	4,896,840	97.66	4,169,777	92.06	4,179,314	94.54	3,978,869	95.98
	C + G	141,690	2.60	91,589	1.97	117,258	2.34	359,513	7.94	241,579	5.46	166,445	4.02
Di-	A + T	2,704,043	65.64	2,243,219	64.01	2,443,975	63.14	2,351,017	63.24	2,227,712	62.59	2,250,023	62.90
	C + G	1,415,455	34.36	1,261,045	35.99	1,426,813	36.86	1,366,875	36.76	1,331,518	37.41	1,327,365	37.10
Tri-	A + T	860,866	40.11	801,886	40.71	845,173	40.42	715,424	42.31	704,848	42.68	703,524	42.21
	C + G	1,285,508	59.89	1,167,953	59.29	1,245,725	59.58	975,508	57.69	946,589	57.32	963,021	57.79
Tetra-	A + T	645,977	72.44	583,242	73.07	673,707	72.93	673,996	73.68	667,142	74.03	730,252	74.86
	C + G	245,743	27.56	214,914	26.93	250,101	27.07	240,760	26.32	233,990	25.97	245,272	25.14
Penta-	A + T	973,912	60.68	893,455	60.93	985,454	61.36	926,318	61.42	948,015	61.25	941,497	61.42
	C+ G	630,973	39.32	572,950	39.07	620676	38.64	581,747	38.58	599,765	38.75	591,298	38.58
Hexa-	A + T	27,274	54.97	19,755	62.20	29,055	57.75	31,488	61.00	32,319	59.88	23,257	57.94
	C + G	22,340	45.03	12,003	37.80	21,255	42.25	20,130	39.00	21,657	40.12	16,883	42.06
Total	A + T	10,528,596	73.78	9,103,265	73.27	9,874,204	72.84	8,868,020	71.44	8,759,350	72.19	8,627,422	72.27
	C + G	3,741,709	26.22	3,320,454	26.73	3,681,828	27.16	3,544,533	28.56	3,375,098	27.81	3,310,284	27.73

^a^ The numbers of nucleotides in SSRs are listed. For example: the total of the mononucleotide SSRs is 5,458,214 bp, of which 5,316,524 bp have A+T and 141,690 bp have C+G.

^b^ For each repeat type, the percent of A + T and C + G are shown.

**Fig 2:**
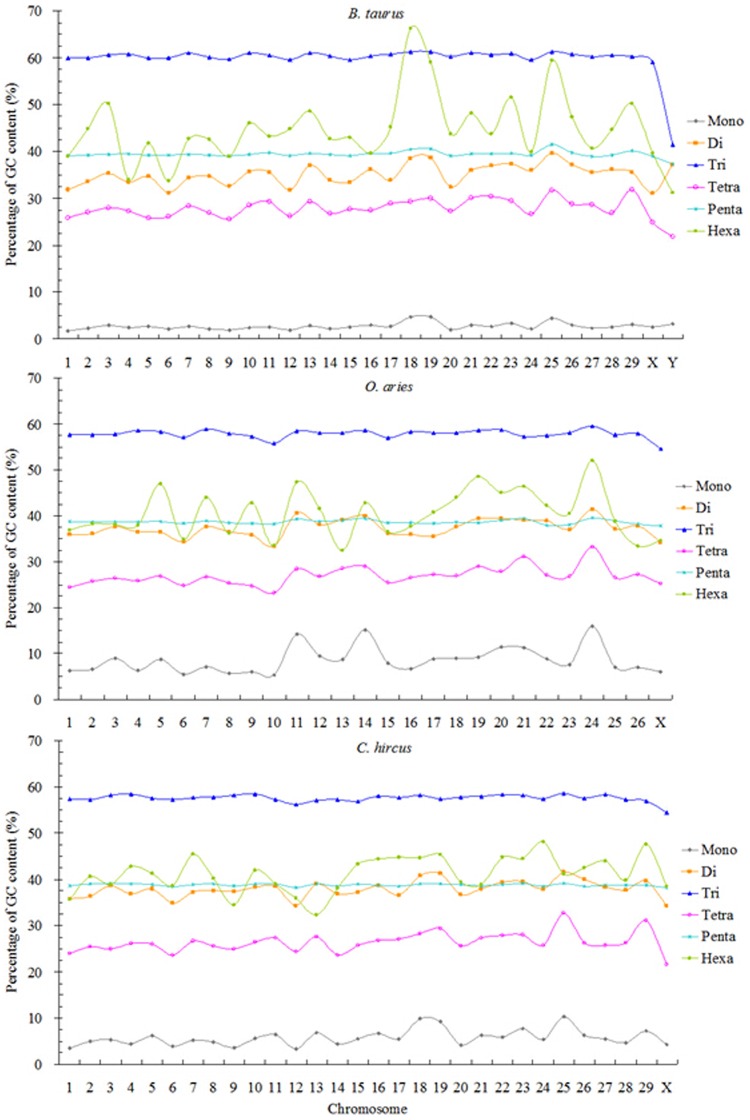
The GC-content of perfect SSRs in all chromosomes of *B*. *taurus*, *O*. *aries*, and *C*. *hircus*.

The GC-content of perfect SSRs was analyzed in all chromosomes of *B*. *taurus*, *O*. *aries*, and *C*. *hircus*, and the results are shown in Fig 2. From the results we can know that except for the chromosome 18 and Y in *B*. *taurus*, trinucleotide SSRs had the most GC-content (over 54.43%) and the least was in the mononucleotide SSRs in any chromosome of the three genomes. SSRs number of all chromosome in the *B*. *taurus*, *O*. *aries*, and *C*. *hircus* is closely positive correlated with chromosome sequence size (Pearson, *r* > 0.980, *p* < 0.01) and significantly negative correlated with GC-content (Pearson, *r* < -0.638, *p* < 0.01). Relative abundance and density in all chromosome of the *B*. *taurus*, *O*.*arie*, and *C*. *hircus* were significantly negatively correlated with GC-content (Pearson, *r <* −0.333, *p* < 0.05) and but not significantly correlated with chromosome sequence size (Pearson, *r* < -0.185, *p* > 0.05). The fluctuation range of GC-content in tri- and pentanucleotide SSRs tended to a horizontal line in all chromosomes of the three bovid species, and so was in the mononucleotide SSRs of *B*. *taurus*. There were some differences in the GC-contents of the same di-, tetra- and hexanucleotide SSRs among different chromosomes of the three bovid species, and so was in the same mononucleotide of *O*. *aries* and *C*. *hircus* chromosomes. The GC-content in the di-, penta- and hexanucleotide SSRs overlap and interweave in all chromosomes of the three species. The percentage sum of GC-content plus AT-content is equal to 100%, from Fig 2 we can know that the AT-contents of mono- to hexanucleotide SSRs were distributed in all chromosomes of the three bovid species.

### 3.4 The distribution of perfect SSRs in the chromosomes of *B*. *taurus*, *O*. *aries*, and *C*. *hircus*

**Fig 3:**
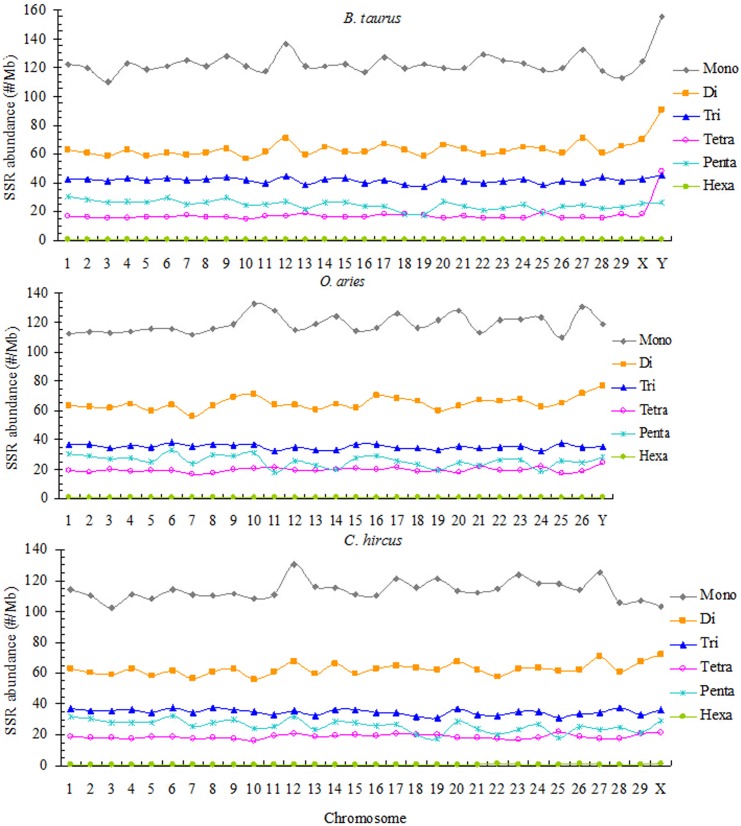
The distribution of perfect SSRs in all chromosomes of the *B*. *taurus*, *O*. *aries*, and *C*. *hircus*.

The relative abundances of the same nucleotide SSR type show highly similarity in all chromosomes of *B*. *taurus*, *O*. *aries*, and *C*. *hircus* (Fig 3). In the relative abundance of all chromosomes of these three bovid species, mononucleotide was the most abundant, followed by the pattern: perfect di- > tri- > penta- > tetra- > hexanucleotide SSRs. The relative overall mono- to tetranucleotide SSR abundances were higher in the *B*. *taurus* Y chromosome than in its autosomes and X chromosome. The relative pentanucleotide SSR abundances was higher in the Y chromosome of *B*. *taurus* than in its autosomes and X chromosome except for chromosome 1, 2, 4, 6, 9 and 12. It's roughly equivalent to the same nucleotide SSRs abundance in the autosomes of *B*. *taurus*. Dinucleotide SSRs abundance were higher in the *C*. *hircus* X chromosome than in its autosomes and so was in the *O*. *aries* Y chromosome than in its autosomes. It is almost equal to the abundance in the same tri-, tetra- and hexanucleotide SSRs of the *C*. *hircus* and *O*. *aries* autosomes. Our analysis revealed that the fluctuations of relative abundance were within a narrow range in all chromosomes of the three bovid species. The relative abundance of mononucleotide SSRs in all chromosomes of *B*. *taurus*, *O*. *aries*, and *C*. *hircus* were mainly concentrated in the 123.73 /Mb, 118.93 /Mb, and 113.58 /Mb, respectively; dinucleotide SSR were mainly concentrated in the 63.59 /Mb, 65.00 /Mb, and 62.57 /Mb, respectively; trinucleotide SSR were mainly concentrated in the 41.53 /Mb, 35.14 /Mb, and 34.81 /Mb, respectively; tetranucleotide SSR were mainly concentrated in the 17.71 /Mb, 19.29 /Mb, and 18.81 /Mb, respectively; pentanucleotide SSR were mainly concentrated in the 24.70 /Mb, 25.58 /Mb, and 25.88 /Mb, respectively; hexanucleotide SSR were mainly concentrated in the 0.55 /Mb, 0.69 /Mb, and 0.73 /Mb, respectively.

## Discussion

### 4.1 Diversity of microsatellite distribution in the bovid genomes

In this study, we used MSDB to scan the recently assembled *B*. *taurus*, *B*. *mutus*, *Bu*. *bubalis*, *O*. *aries*, *C*. *hircus*, *P*. *hodgsonii* genomes for microsatellites of 1–6 bp. To compare our results, we performed a similar analysis of these bovid genomes using the same bioinformatics tool and search parameters. Clearly, these data provide evidence of similarity patterns of SSRs distribution in bovid genomes, indicating that the particular contribution of these SSRs to the genome of the six bovids may be the rule for other bovid species. Mononucleotides SSRs were the most abundant repeat type, accounting for 43.01%– 45.33% of all of the SSRs, followed by the pattern: di- > tri- > Penta- > tetra- > hexanucleotides SSRs in the study. Eukaryotic genomes are characterized by the prevalence of mononucleotide repeats over other nucleotide repeat classes [24]. Mononucleotide repeats are the most abundant class of SSRs in all the human chromosomes [25], *Volvariella volvacea* and *Agaricus bisporus* [26]. However, dinucleotide repeats are the most abundant SSRs in rodents [5] and majority of the dicot species [27]. Trinucleotide repeats are the most abundant SSRs in *Neurospora crassa* [28], *Cyanidioschyzon merolae*, *Thalassiosira pseudonana* [24], *Coprinus cinereus*, *Schizophyllum commune*, *Pleurotus ostreatus* [26] and *Eremothecium gossypii* genomes [24], which could indicate their structural similarity with prokaryotes. Previous research has shown that hexanucleotide repeats are the most abundant SSRs in the coding regions of eukaryotes [25]. Here, hexanucleotide SSRs appeared significantly underrepresented, with as few as 0.15%– 0.29% of the total number of SSRs in the bovid species. In contrast, tetranucleotide SSRs were less abundant than pentanucleotide SSRs in the study. It might be due to positive selection of even-number motif repeats relative to odd-number motif repeats. Alternatively, there could be a more passive reason, namely that even-number motif repeats might be favored to accumulate and/or to be maintained [25]. Further studies will be required to test these possibilities.

The smaller motifs were predominant in each genome, as motif length increases, the occurrence decreases. This trend has been observed for a range of organisms [23]. Among mononucleotide repeat type, the motif (A/T)_n_ were predominant, while (C/G)_n_ repeats were rare in these bovid genomes. Also, the (A/T)_n_ motif was the most frequent mononucleotide repeats in *A*. *bisporus*, *V*. *volvacea*, *C*.*cinereus*, *P*. *ostreatus* [26], *Caenorhabditis elegans*, *Brugia malayi*, *Meloidogyne hapla* [14], and *Carlavirus* [29], whereas the (C/G)_n_ motifs were most frequent in the *S*.*commune* [29], *Meloidogyne incognita* and *Pristionchus pacificus* [14] genomes. Among the dinucleotide SSRs of these bovids, the (AC)_n_ motif seem to be predominant compared with other motifs, while (CG)_n_ were extremely rare and all present in these Bovidae species. Also, (AC)_n_ motif was predominant in human beings [25] and *Carlavirus* [29], and (AG)_n_ motifs are the most abundant in *Magnaporthe grisea*, *Ustilago maydis* [23,28], *Camellia sinensis* L. [30], nematodes [14], insects [31] and other invertebrates [32], while the (CG)_n_ repeats were extremely rare. This is especially interesting because (CG)_n_ motifs were also rare in human beings, *Drosophila melanogaster*, *C*. *elegans*, *Arabidopsis thaliana* [32], *Brassica rapa* [33], yeast [32], and fungi [23, 28].

Our study showed that the occurrence of (AC)_n_ motif was nearly 261.03 times on average as abundant as the (CG)_n_ motif in the bovid genomes (Table 2). The lower frequencies of (CG)_n_ motifs can be explained on the basis of A/T richness and the relative difficulty of strand separation for C≡G compared to A≡T and other tracts [6]. In the same way, trinucleotide SSRs were dominated by CG-rich motifs, with (AGC)_n_ and (ACG)_n_ being always present in the most common motifs and (CCG)_n_ being forever existing in the least frequent motif in the bovid genomes investigated. Previous study revealed that the (AAG)_n_ motif predominated in *Potyvirus* [6], *Aspergillus nidulans*, *Cryptococcus neoformans*, *Encephalitozoon cuniculi*, *Saccharomyces cerevisiae* [23], *C*. *elegans* [14], *Serpula lacrymans* [34] and the (AAT)_n_ motif in *M*. *hapla*, *P*. *pacificus*, *B*. *malayi* [14] and *Schizosaccharomyces pombe* [23], and the (ACG)_n_ motif in *Ganoderma lucidum*, *Coprinopsis cinerea*, *Laccaria bicolor*, *Postia placenta* [34] and *U*.*maydis* [23], whereas (CCG)_n_ motif was the most frequent in *Phanerochaete chrysosporium* [34] and *M*. *grisea* [23], the (AAC)_n_ motifs are the most frequent in *M*. *incognita* [14] and *N*. *crassa* [23]. The (AACTG)_n_ and (AGTTC)_n_ motifs were the two most frequent tetranucleotide repeat units in these species and none of these single hexanucleotide motifs appeared to be shared by the six bovid species. The (AAACAA)_n_ motif was most frequent in *B*. *mutus*, *Bu*. *bubalis*, *O*. *aries*, and *C*. *hircus*, whereas the (AAAGTG)_n_ motif was most frequent in *B*. *taurus* and *P*. *hodgsonii*. Overall, the diversity of SSRs motifs gave each of the six bovid species a similarity pattern of SSRs distribution, suggesting that they can be nearly phylogenetic relationships. Conversely, none of the most frequent di- to hexanucleotide motifs contains exclusively Cs or Gs. The relative abundances of the same SSRs motifs show great similarity in the Bovinae species and so is in the Caprinae species. Indeed, such a consistency in the study may be considered as a strong indication of the robustness of the global analysis.

### 4.2 The GC-content in all analyzed SSRs

It has been reported that the level of GC-content may play some important roles in the entire genome. Indeed, the (G)_n_ mutants in the thymidine kinase (TK) gene (tk) was reported to be related with the reactivation of herpes simplex virus [35]. The high GC-content repeats have also been reported to be related to some diseases in human and the pathogenesis of some microorganisms. For example, fragile X mental retardation-1 (FMR-1) alleles with the (CGG)_n_ repeats were associated with neurodegeneration [36] and ovarian insufficiency [37]. FRA12A mental retardation resulted from the expansion of a large (CGG)_n_ tract in the 5′ UTR of the DIP2B gene [38]. The (G)_n_ repeats in membrane protein-gene pmp10 of *Chlamydophila* (*Chlamydia*) *pneumoniae* was involved in virulence and pathogenesis of *Chlamydia* [39] and the (C)_n_ in outer membrane proteins was involved in the pathogenesis of *C*. *pneumoniae* [40]. Long SSR with 5–11bp motif (SSR^5–11^) were more common in GC-rich genomes, and large genomes tend to be GC-rich, and the weak correlation between Long SSR^5–11^ counts and GC-content may arise as an artifact of correlations of both with the genome size [4]. Interestingly, GC-rich SSRs were generally more difficult to expand in these PCR experiments, seemingly agreeing with our observation. There was a negative correlation between the GC-content of the flanking regions of SSRs and its polymorphism [41], which might be valuable in choosing SSRs markers. This may be due to the preponderance of motif repeats with low GC-content and SSRs frequently constitute genomic regions of low Tm.

Data-mining of 26 completed genomes showed that SSRs with low GC-content were predominant in most eukaryotic genomes [24]. This trend also emerged from our survey, with the majority of the most frequent SSRs motifs from bovids being AT-rich. The (A/T)_n_ motifs were significantly more prevalent than the (G/C)_n_ motifs in each complete bovid genome, whose difference could be explained by the AT-content being only notably higher than GC-content in each of the analyzed sequences. Trinucletide SSRs had high GC-content in monocot genomes [42], which was consistent with our study. The GC-content of SSRs in different coding regions was different. For example, GC-content of those reverse repeat regions (RS and RL) was significantly higher than that in unique long and unique short regions (UL and US) in *Herpes simplex* virus type 1 (HSV-1) [43]. Also, the GC-content of SSRs in RS- and RL-coding regions is significantly higher than that in UL- and US-regions. This could be due to the different mutational pressure in different coding regions [44]. The GC-content has been shown to covary with genomic properties such as regulated replication or expression timing [45–46], DNA bendability [47] and ability to B–Z transition [48]. The (CCG)_n_ repeats can form secondary structures (hairpin-like) that escape DNA repair in yeast [49]. The (CCG)_n_ repeats which were rich in HSV-1 genome were exhibited considerable hairpinforming and quadruplex-forming potential [10]. Therefore, the high GC-content in genome may affect the genome structure, especially the high GC-content in SSRs.

### 4.3 Distributional difference of SSR abundance, density and GC-content on different chromosomes

Sex chromosomes have been found to differ in SSRs density from autosomes in many eukaryotes. Human, rat [50], and mouse [51] X chromosomes were found to have a lower abundance of SSRs compared to autosomes, whereas the reverse was the case for dinucleotide SSRs in the *Drosophila* X chromosome [52]. The Z chromosome of *Bombyx mori*, equivalent to the X chromosome of mammals and *Drosophila*, had a higher trinucleotide SSRs density in the Z chromosome than in its autosomes [53]. In the mon- and dinucleotide SSRs of *B*. *taurus*, *C*. *hircus*, and *O*. *aries*, all chromosomes had the highest abundance and density of (A)_n_ and (AC)_n_ motifs. The autosomes and X chromosome of these three bovid species had the highest abundance and density of (ACG)_n_ and (AGC)_n_ motifs in trinucleotide SSRs, whereas the *B*. *taurus* Y chromosome had the highest abundance and density of (AAC)_n_ motifs. Also, the autosomes and X chromosome of these species all had the highest abundance and density of (AAAT)_n_ motifs in tetranucleotide SSRs, whereas the Y chromosome of *B*. *taurus* had the highest abundance and density of (AAAC)_n_ and (AAAG)_n_ motifs. The *B*.*mori* Z chromosome had a higher density of (ATT)_n_ repeats compared to the autosomes, and its sequences contained very few tetra- and hexanucleotide repeats and were devoid of pentanucleotides [53]. In these three Bovidae species, all chromosome sequences also contained very few hexanucleotide SSRs.

It is almost equal to the GC-contents in the same tri- and pentanucleotide SSRs of the *B*. *taurus*, *O*. *aries*, and *C*. *hircus* autosomes, whereas the reverse was the case for the same mono-, di-, tetra- and hexanucleotide SSRs in its autosomes and sex chromosomes (Fig 2). The GC-contents of tri- to hexanucleotide SSRs were less in the X and Y chromosomes of *B*. *taurus* than that in its autosomes. Trinucleotide SSRs had the most GC-content except for the chromosome 18 and Y in *B*. *taurus*, and the least was in the mononucleotide SSRs in the chromosomes of the three bovid genomes. The GC-contents of tri- and hexanucleotide SSRs were less in the X chromosomes of *O*. *aries* than that in its autosomes. And the GC-contents of di- to pentanucleotide SSRs were less in the X chromosomes of *C*. *hircus* than that in its autosomes (Fig 2).

## Conclusions

The mononucleotide SSRs were the most abundant, followed by the pattern: di- > tri- > penta- > tetra- > hexanucleotide SSRs. Generally, the number of SSRs, relative abundance, and relative density of SSRs decreased as the motif repeat length increased in each species of the Bovidae. The most GC-content was in trinucleotide SSRs and the least was in the mononucleotide SSRs in the six bovid genomes. The GC-contents of tri- and pentanucleotide SSRs display a great deal of similarity among different chromosomes of *B*. *taurus*, *O*. *aries*, and *C*. *hircus*. The SSR number of all chromosomes in the *B*. *taurus*, *O*.*arie*, and *C*. *hircus* is closely positively correlated with chromosome sequence size and significantly negatively correlated with GC-content. Relative abundance and density of SSRs in all chromosomes of the three species were significantly negatively correlated with GC-content and but not significantly correlated with chromosome sequence size. These data provided evidence for similarity patterns of SSR distributions in the six bovid species, which indicated that the particular contribution of their SSRs may be the rule for other bovids.

## Supporting Information

S1 Table The most and least frequent motifs for different length shared by all chromosomes of the *B*. *taurus*, *O*. *aries*, and *C*. *hircus*. (RAR)
